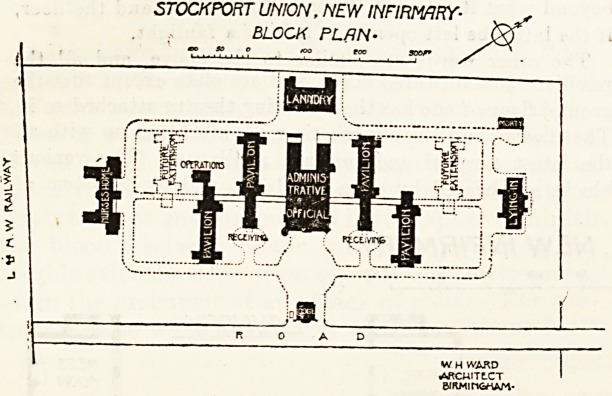# New Workhouse Infirmary at Stepping Hill, Stockport

**Published:** 1906-09-22

**Authors:** 


### HOSPITAL ADMINISTRATION.

### CONSTRUCTION AND ECONOMICS.

### NEW WORKHOUSE INFIRMARY AT STEPPING HILL, STOCKPORT.

(*Continued from page* 430.)

Crossing the corridor the ground-floor ward of the pavilion is reached. On the left there is a small "separation" ward, presumably for one bed, and on the right hand are the nurses' duty-room, having an inspection window looking into the large ward, a pantry and a linen-room. The door of the large ward is placed at the end of the short passage which leads from the main corridor and runs between the "separation" ward and the nurses' room. The ward is 72 ft. long and 24 ft. wide, and contains 24 beds. Each bed has, therefore, 72 ft. of floor space, and 6 ft. of wall space; and, assuming a ceiling height of 12 ft., each patient would have 864 cubic feet of air space. In a general hospital this would be considered totally insufficient; but the cases usually under treatment in a workhouse infirmary either do not require, or are not thought to require, so much space; but after all that has been said about cubic space it is certain that far more will depend on the medical officer's idea of the influx of fresh air than on the mere size of a ward and the number of beds. In this ward the beds are placed in pairs between the windows, which again would not be permissible in a modern general hospital. At the further end of the ward are the sanitary annexes, and these are not sufficiently cut off from the ward by cross-ventilated passages. In fact the passages are not cross-ventilated at all, although there is cross-ventilation through the annexes themselves. There is an escape staircase and a good verandah. As is so often found in hospitals, the single-bedded, or separation, ward has no cross-ventilation beyond what it may get through the window and the door, if the latter be left open or if it have a fanlight.

The other wards are similar to the above, and all the pavilions contain three floors, and are alike except that the ground floor of one has the operating theatre attached to it. The theatre and the anæsthetic room are fitted up with all the latest surgical and sanitary appliances. The various blocks are heated by open fireplaces, and by a system of radiators. This combination is the best known, especially if the fires be made to supply the most of the heat and the radiators kept in reserve for very cold weather.

The nurses' home is a three-story building, consisting of a centre and wings. The entrance is central and has a verandah on each side. A corridor passes from wing to wing connecting these with the recreation-room and the mess-room which are centrally placed. The right-hand wing contains the kitchen, larder, lavatory, and closets. On the other side of the corridor is the staircase, and close to it is a passage running at right angles to the main corridor, which passage gives access to four bedrooms. The other wing is similar save that the head nurse's room takes the place of the kitchen. The upper floors are similar

STOCKPORT UNION. NEW INFIRMARY.

in arrangement, excepting that the space above the recreation and mess-room is divided into bedrooms.

The maternity block also consists of a centre and wings. The former has the entrance hall, duty-room, bath-room, and labour-rooms. The wards occupy the whole of the wings, and each ward contains six beds. This block is exceedingly well planned in every way. The position of the sanitary annexes is good, and these annexes are properly cut off from the main by cross-ventilated lobbies.

The mortuary block has room for ten bodies, and there is a separate division for post-mortem examinations, this part being fitted up in the latest manner.

The laundry is placed at the back of the parallelogram. It is very compactly arranged, and contains machinery of the best pattern.

The external walls of the infirmary are faced with red bricks, and the window-sills and heads are of stone. The floors of the pavilions are laid down with oak blocks; but otherwise they are of fireproof construction. The main corridor is floored with granolithe. The engineering works comprise cooking apparatus, boilers, gas and electric plant, hydrants, telephones, etc., and are carried out in the most complete and approved principles throughout. The cost of the whole buildings and adjuncts works out at £130 a bed, which must be looked upon as moderate nowadays, and must compare favourably with other infirmaries considering the way this one has been carried out.

The architect was Mr. H. Ward, of Birmingham, and the contractor was Mr. D. Eadie, of Stockport.

## Figures and Tables

**Figure f1:**
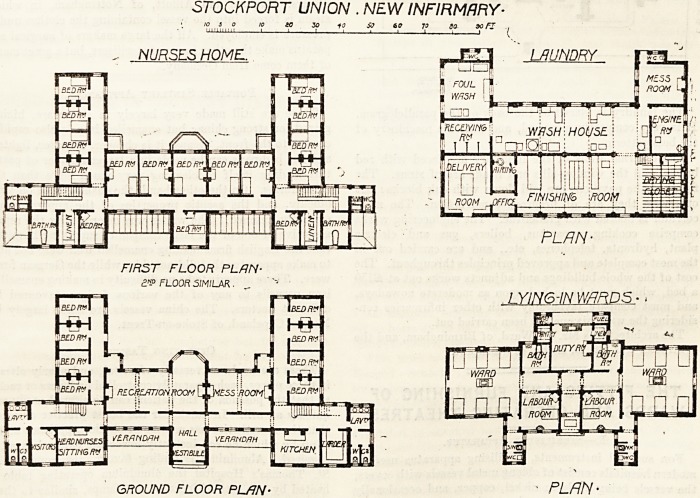


**Figure f2:**